# Quantifying Risk Factors for Human Brucellosis in Rural Northern Tanzania

**DOI:** 10.1371/journal.pone.0009968

**Published:** 2010-04-01

**Authors:** Kunda John, Julie Fitzpatrick, Nigel French, Rudovick Kazwala, Dominic Kambarage, Godfrey S. Mfinanga, Alastair MacMillan, Sarah Cleaveland

**Affiliations:** 1 Muhimbili Medical Research Centre, National Institute for Medical Research, Dar-es-Salaam, Tanzania; 2 Division of Ecology and Evolutionary Biology, University of Glasgow, Glasgow, United Kingdom; 3 Moredun Research Institute, Edinburgh, United Kingdom; 4 Institute of Veterinary, Animal and Biomedical Sciences, Massey University, Palmerston North, New Zealand; 5 Sokoine University of Agriculture, Morogoro, Tanzania; 6 Veterinary Research Directorate, Department of Environment, Food and Rural Affairs, London, United Kingdom; The Kenya Medical Research Institute, Kenya

## Abstract

**Background:**

Brucellosis is a zoonosis of veterinary, public health and economic significance in most developing countries. Human brucellosis is a severely debilitating disease that requires prolonged treatment with a combination of antibiotics. The disease can result in permanent and disabling sequel, and results in considerable medical expenses in addition to loss of income due to loss of working hours. A study was conducted in Northern Tanzania to determine the risk factors for transmission of brucellosis to humans in Tanzania.

**Methods:**

This was a matched case-control study. Any patient with a positive result by a competitive ELISA (c-ELISA) test for brucellosis, and presenting to selected hospitals with at least two clinical features suggestive of brucellosis such as headache, recurrent or continuous fever, sweating, joint pain, joint swelling, general body malaise or backache, was defined as a case. For every case in a district, a corresponding control was traced and matched by sex using multistage cluster sampling. Other criteria for inclusion as a control included a negative c-ELISA test result and that the matched individual would present to hospital if falls sick.

**Results:**

Multivariable analysis showed that brucellosis was associated with assisted parturition during abortion in cattle, sheep or goat. It was shown that individuals living in close proximity to other households had a higher risk of brucellosis. People who were of Christian religion were found to have a higher risk of brucellosis compared to other religions. The study concludes that assisting an aborting animal, proximity to neighborhoods, and Christianity were associated with brucellosis infection. There was no association between human brucellosis and Human Immunodeficiency Virus (HIV) serostatus. Protecting humans against contact with fluids and tissues during assisted parturition of livestock may be an important means of reducing the risk of transferring brucellosis from livestock to humans. These can be achieved through health education to the communities where brucellosis is common.

## Introduction

In sub-Sahara African countries, many zoonoses are poorly controlled in both livestock and human populations, thus endangering poor people's livelihoods by affecting their livestock and compromising their health and survival [1]. Zoonoses also cause great economic losses to poor people particularly in the rural areas of sub-Sahara African countries [2]. Brucellosis is a zoonosis of veterinary, public health and economic significance in most developing countries [3]. Human brucellosis is a severely debilitating disease that requires prolonged treatment with a combination of antibiotics leaving permanent and disabling sequel, and results in considerable medical expenses in addition to loss of income due to loss of working hours [4], [5], [6]. In livestock, brucellosis results in reduced productivity, abortions and weak offspring and is a major impediment for trade and export. Almost all domestic species can be affected. Thus, its prevention, control and eradication are a major challenge for public health programmes [4], [5]. The disease has been eradicated in a number of countries, including the UK, since 1980–81. Even in these countries however, human infections are still encountered as the occasional case arising from endemic areas [3].

Where brucellosis exists in sheep and goats, it causes the greatest incidence of infection in humans [3]. Most human cases involving field strains of *Brucella* species can be traced to domestic animals, and the prevalence of disease in humans reflects its occurrence in livestock reservoirs. Commonly, *B. abortus* and *B. suis* infections are associated with certain occupational groups, including farm workers, veterinarians, and meat-packing employees [7]. Transmission through consumption of contaminated dairy products is the route that has been well documented in many parts of the world [8]. Unpasteurized milk and processed dairy foods from infected animals have been considered a source of infection for the general population, and infected carcasses as a source of infection for workers in the meat-packing industry. Veterinarians may acquire brucellosis from assisting births in infected livestock, as well as through inadvertent exposure to vaccines [7]. Veterinarians, laboratory staff, and workers based in meat plants were found to be at increased risk of exposure in Ireland and Spain [9], [10]. In Burundi, the prevalence of positive serology was found to be significantly higher in professionally at risk people than in people consuming contaminated food [11]. Airborne transmission of bacteria to humans has also been documented in clinical laboratories and abattoirs [12].

Contact with contaminated products of conception from animals has been shown to be an important factor in the transmission of brucellosis to humans [7]. In Kyrgyzstan, brucellosis was shown to be associated with exposure to aborted farm animals in the household and consumption of home-made milk products obtained from bazaars or neighbors [13]. Touching calves or placentas that were infected with the *Brucella* species was found to be associated with brucellosis transmission during cattle birth in Korea [14]. Similar findings were obtained in Greece, Chad and Saudi Arabia [15], [16], [17] where products of conception, especially the placenta were found to be a risk factor for brucellosis transmission.

The economy of Tanzania depends largely on agriculture, of which livestock forms an integral part [18]. As the economic future of the country lies mainly in agricultural development, diseases with considerable effect on livestock productivity and human health such as brucellosis should be controlled by all possible means [19]. A large proportion of Tanzania's population lives in rural areas with high levels of contact with livestock and their products. Brucellosis occurs widely in livestock keeping populations in Tanzania [20]; where a 7.7% prevalence has been reported in northern Tanzania [21]. However, very little data is available on specific risk factors for human infection in different livestock-keeping communities in Tanzania. The objectives of the current study were to explore factors responsible for transmission of brucellosis to humans in Arusha and Manyara regions and identify potential preventive measures to minimize the transmission of the disease from animals and their products.

## Methods

### Ethics

The study protocol was peer reviewed and cleared for ethics by the Medical Research Co-ordinating Committee of the National Institute for Medical Research. Verbal and written consents were also sought from all participants before being involved with the study.

### Study area

The study was conducted in Arusha and Manyara regions in the northern Tanzania. The regions comprice the majority of the nomadic livestock keeping communities in Tanzaia. The major ethnic groups in the regions include the Maasai, Mbulu (Iraqw), Barbaig, Fyomi and Sonjo. Maasai and Barbaigs are primarily livestock keepers, practicing traditional pastoralism and following a semi-nomadic lifestyle. The predominant form of land-use among the other ethnic groups is agropastoralism with people keeping livestock, but also growing crops for subsistence. A study conducted by Cox in 1966 [22] among the nomadic communities indicated that living in close contact with livestock and traditional practises exposes people who practice nomadic type of life-style to the high risk of brucellosis. Nomads move with their cattle long distances and in so doing, the animals are liable to acquire infection from a wide range of pastures. The infection is then easily transmitted to humans due to different traditional practices.

**Figure 1:**
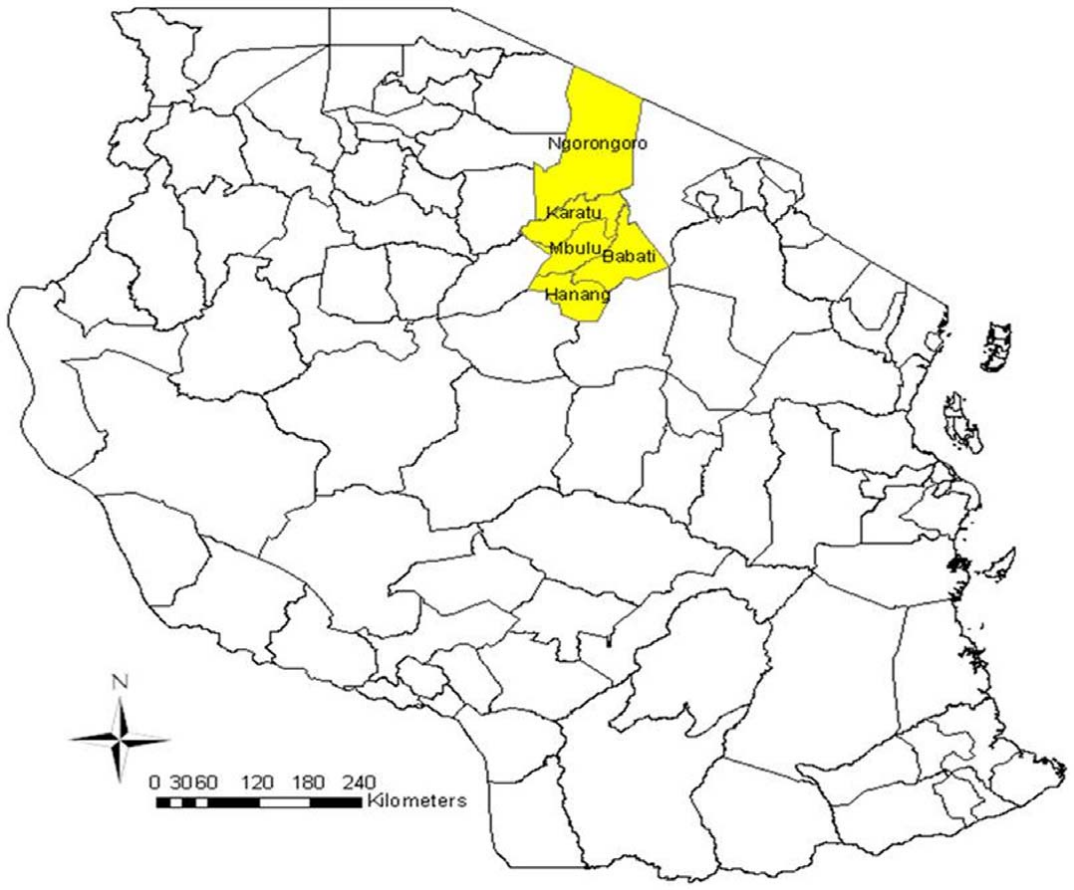
The map of Tanzania showing the study area.

Hospitals involved with the study included Babati and Dareda hospitals in Babati, Mbulu and Hydom hospitals in Mbulu, Katesh hospital in Hanang, Karatu Lutheran hospital in Karatu and Endulen and Wasso hospitals in Ngorongoro (Figure 1). The majority of patients in the study area go to district or designated district hospitals than dispensaries because the hospitals are more equipped and staffed than dispensaries.

### Study design, inclusion criteria and blood sampling

This was designed as a matched case-control study. All patients who presented to the selected hospitals between July 2002 and June 2003 with febrile illnesses were enrolled into the study. There was no age limit but any patient who did not belong to the districts in the study area was given appropriate treatment but was not included in the study. Blood was sampled and tested for brucellosis at the hospitals using the Rose Bengal Plate Test (RBPT) [23] and patients were treated according to laboratory results and clinical symptoms found. For each blood sample, an aliquot was stored for c-ELISA test [24] at the Veterinary Laboratory Agencies (VLA) in the UK and for HIV using Vironostica Uniform II *Plus* O, at the Muhimbili University College of Health Sciences in Dar es Salaam.

Based on the c-ELISA test as a confirmatory test for brucellosis, all patients who presented to the selected hospitals, who tested positive for brucellosis using the c-ELISA test conducted at the VLA and showing at least two of the following clinical features: headache, recurrent or continuous fever, sweating, joint pain, joint swelling, general body malaise or backache, were defined as brucellosis cases. For every case, a community-based control was selected randomly using a multi-stage cluster sampling method. A control was an individual in the same district as the case, having a negative brucellosis serological result by the c-ELISA test, matched by sex and coming from a hospital-going household.

### Questionnaire data collection and household blood sampling

Cases and controls were visited at their homesteads for household blood sampling and questionnaire data collection on potential risk factors for brucellosis, including types of livestock they keep, handling of livestock and their products, consumption of animal products, history of brucellosis in the household, level of education, socio-economic status, personal particulars such as tribe, religion, location of households from nearest neighbour and from village centre etc. Samples of blood were taken from all members of households of cases and controls visited. Interviews were conducted by the principal investigator in Swahili, the language commonly used in the area. In each household, livestock (cattle, sheep and goats) were also sampled for brucellosis testing. In the field, samples of blood from livestock were tested for brucellosis using RBPT and an aliquot was stored for c-ELISA test at the VLA. The number of livestock to be bled was determined by using the power of 80% with 95% confidence and prevalence of brucellosis of 5% to detect infection in a herd [25].

### Data analysis

A conditional logistic regression was performed to analyse case-control data using Egret for Windows version 2.0 (Cytel Software Corporation, Cambridge Massachusetts). The univariable relationships between all independent variables and brucellosis were estimated by including them individually in a model with brucellosis serological result as the dependent variable. Forty-four risk sets were obtained from the cases and controls studied. Using risk sets as a matching variable, and cases and controls as outcome variables, a number of models were fitted to test their significance as risk factors for a brucellosis positive serological result.

Since a few samples were available for HIV testing, the univariate analysis using HIV results was carried out separately using Epi-info 6 and the results were interpreted separately without being included in the multivariate analysis. All the 95% confidence intervals were calculated using Epi Info 6 software (CDC, Antanta, Georgia).

Multivariable models were created by a backward stepwise procedure using Egret for windows. Variables that had a p value of ≤0.2 from the univariable analysis were considered for inclusion in the final models. Values were retained if on their removal there was a significant increase of the residual deviance of the model with likelihood ratio statistics (LRS) of p>0.05 and they were removed from the model if they caused an insignificant increase or decrease in the residual deviance with LRS of p<0.05.

## Results

Of the 98 cases identified in hospitals, 44 were available for follow-up (Table 1). Of the 44 cases, 25 were males and 19 females. Four cases died before a follow-up was conducted; these included two patients from Hanang district, one from Karatu district and one from Ngorongoro district. A total of 55 controls were followed-up (Table 1) during the study period, of these 29 were males and 26 females. The mean age for the cases was 36.4 and for controls 36.3 with standard deviations of 17.8 and 17.3 respectively. *Brucella* seroprevalence in humans based on the c-ELISA test conducted at the VLA was 7.7%. The prevalence of brucellosis among cases and controls livestock was 4.6%, 3.4% and 3% for goats, sheep and cattle respectively. Infection in humans and that in flocks of livestock was compared at household, village and district levels. There was a significant association between prevalence of brucellosis in humans and prevalence of brucellosis in goats at district level (p<0.05).

**Table 1:** Cases and controls followed-up.

	Babati	Hanang	Karatu	Mbulu	Ngorongoro	Total
Case	11	24	1	1	6	44
Control	12	34	1	1	8	55
**Total**	**23**	**58**	**2**	**2**	**14**	**99**

### Univariable analysis of risk factors for human brucellosis

Univariable relationships between independent variables and a brucellosis cases result are shown in Table 2. The likelihood of becoming a brucellosis case increased with any animal abortion in the herd, history of household member suffering from brucellosis and decreasing distance to the nearest neighbour, irrespective of neighbour's serostatus. The likelihood of brucellosis also increased with Christian religion, households where a goat had aborted, involvement in preparing meat, involvement in assisting aborting livestock and a household that was of a middle socio-economic status.

**Table 2:** Univariable relationships between independent variables and brucellosis cases.

Variable	Coefficient	Standard error	p-value	Odds ratio	95% ConfidenceUpper Lower		LRS p- value
Distance to the nearest neighbour's house-continuous	−0.01	0.004	0.02	0.99	0.98	0.99	0.007
Christian religion	2.81	0.78	0.07	4	1.03	13.46	0.04
Any member suffered from brucellosis	1.72	0.79	0.03	5.61	1.19	26.28	0.01
Abortion of any animal	1.27	0.46	0.006	3.55	1.43	8.80	0.003
Goat aborted	1.07	0.44	0.01	2.90	1.23	6.86	0.009
Involved in assisting abortion	1.49	0.65	0.02	4.46	1.25	15.92	0.009
Prepared any meat	0.96	0.48	0.04	2.62	1.02	6.74	0.03
Economic status- Low	0.80	0.71	0.26	2.24	0.55	9.05	
Economic status- Middle	2.51	0.95	0.008	12.31	1.92	78.79	
Economic status- High	0.16	1.06	0.88	1.17	0.15	9.39	0.008

### Multivariable analysis

Three variables were included in the final model (Table 3). Brucellosis was significantly associated with involvement in assisting with abortion, with proximity to the nearest neighbor and with the Christian religion (likelihood ratio statistics p<0.001).

**Table 3:** Multivariable relationship between independent variables and brucellosis.

Likelihood ratio test	Coefficient	Std.Error	p-value	Odds Ratio	95% confidence intervalLowerUpper	
Distance of house to nearest neighbour's house Continuous	−0.02	0.01	0.01	0.98	0.97	0.99
Involved in assisting abortion	2.06	0.84	0.01	7.86	1.51	40.87
Christian religion	1.94	0.98	0.03	3.03	2.11	18.44

### Result of HIV testing

HIV testing using Vironostica Uniform II *Plus* O (bioMérieux bv, Boxtel, The Netherlands) was done on 66 samples, including 37 controls and 29 cases (Table 4). Of the samples tested, two cases tested positive for HIV and three controls (3.1%, 95% CI, 0.2–17.9) tested positive for HIV (OR, 0.84, 95% C I, 0.13–5.39).

**Table 4:** HIV ELISA results of human cases and controls.

	ELISA		
	Positive	Negative	Total
Case	2	27	29
Control	3	34	37
**Total**	**5**	**61**	**66**

## Discussion

This study is the first to examine risk factors for human brucellosis in Tanzania and showed that of the livestock-associated risk factors, brucellosis was strongly associated with assisting aborting livestock. An abortion storm in a herd of livestock is among the common features of brucellosis in livestock [16]. During abortion, large numbers of *Brucella*e are released which may, in turn, cause the infection to other animals in the herd [26]. The finding that contact with livestock during parturition is a strong risk factor for brucellosis is consistent with results from other studies which demonstrate an increased risk in association with assisted parturition [7], [15], [16]. The findings also agree with Young in 1983 [7] who found that persons usually become infected with *Brucella* through direct contact with infected animals or their products. In a study conducted in Greece, human trauma during animal delivery was found to increase the risk for contracting brucellosis [15].

In Chad, a study conducted by Schelling *et al* in 2003 [16] showed that contact with placenta of livestock was highly associated with brucellosis transmission. In Saudi Arabia, assisting animals during parturition w as found to be an important risk factor for brucellosis transmission, but no significant risk associated with other direct (unspecified) animal contact was observed [5]. However, these studies did not link the risks to contacts with products of abortions directly but rather to contacts with products of conception, it is possible that most of these contacts were with products of incomplete term pregnancies which in most cases are due to brucellosis.

In other parts of Africa brucellosis transmission to humans was associated with a wide range of risk factors, but all relate to transmission through direct contact with animals or their products or indirectly through consumption of their products. In Nigeria the highest prevalence (20%) of brucellosis was observed among cattle handlers followed in decreasing order of prevalence by goat rearers (10%), mixed sheep and cattle rearers (9%), mixed sheep and goat rearers (8%), and 4% among each of sheep rearers and non-rearers of animals [9]. The social habit of eating raw meat, e.g. raw liver or other offal with spices (Marrara or umfitfit) was found to be an important epidemiological factor in contracting the disease in central Sudan, the majority of the patients were found to have a combined infection of both *Brucella abortus* and *Brucella mellitensis*. [27]. Although studies conducted in the same regions of Arusha and Manyara showed that eating uncooked meat or meat products was a common practice [28], the current study did not find it to be a risk factor for brucellosis causation.

Distance between households was found to be an important factor in the transmission of brucellosis. The closer the households the greater was the chance of contracting brucellosis, irrespective of the serostatus of the neighbours. In most African communities, neighbours assist each other in conducting different activities. These range from home-based to farm-based duties. Assisting neighbour's animal during parturition, sharing of food stuffs such as milk and other dairy products is also not uncommon in communities visited in Arusha and Manyara regions. It is likely that some of the cases acquired brucellosis while assisting an aborting animal from a neighbour. This finding could explain the lack of association between the human sero-status and that of their livestock in a household established in the current study.

There is no immediate explanation for the association between brucellosis and people belonging to Christian religion. There are many practices however that could be linked to the religious groups in Arusha and Manyara regions that need be taken into consideration and need further investigation as far as the risks for brucellosis are concerned. Animal husbandry, the number of livestock kept, interactions between livestock and humans amongst Christians in comparison to other groups (Muslims or atheists) could be important factors for comparison in the study area.

HIV is known to be a risk factor for zoonoses such as bovine tuberculosis [29]. In the current study no association was observed between HIV sero-status and brucellosis (OR = 0.84). In a study conducted in Kenya by Paul *et al., in* 1995 [30], no association between *Brucella* antibody status and HIV status was established and in a study conducted in Spain by Moreno *et al.,* in 1998 [31], HIV infection was found not to increase the incidence of brucellosis. However, in the current study the number of samples that were available for HIV testing was few and hence the results obtained should be extrapolated with caution.

### Conclusion

While contact with products of conception has been shown to be a risk factor for brucellosis transmission in other places, closeness of households in livestock keeping communities and the social background have not been documented as important risk factors for brucellosis transmission. The current study indicates that health education on ways to prevent brucellosis transmission through contact while assisting animal delivery should be given a priority in Arusha and Manyara regions. Though it may be difficult for each and every farmer to acquire and use protective gloves while assisting animal parturition because of affordability and availability issues, other simple and cheap material such as plastic bags that are easily available in the rural settings could be used to prevent direct contact with the products of conception from livestock that have been shown to have high concentrations of *Brucella*. Other items that can be used include papers and clothes that could be disposed off after a single use. The study also indicates the importance of increasing awareness even to those who don't keep livestock on the potential of acquiring brucellosis from their neighbours' livestock through contact with infected products of conception.
